# Enabling Informed Decisions on Pyrolysis: A Key to Turn the Tide on Plastics Recycling

**DOI:** 10.1021/acssuschemeng.4c09908

**Published:** 2025-06-02

**Authors:** Patritsia Maria Stathatou, Elisavet Anglou, Yuchen Chang, Jacob Sweet, Arvind Ganesan, Natechanok Yutthasaksunthorn, Erin V. Phillips, Nikhita S. Ragam, Omar Isaac Asensio, Sankar Nair, Fani Boukouvala, Carsten Sievers

**Affiliations:** 1 School of Chemical & Biomolecular Engineering, 1372 Georgia Institute of Technology, Atlanta, Georgia 30332, United States; 2 Renewable Bioproducts Institute, 1372 Georgia Institute of Technology, Atlanta, Georgia 30332, United States; 3 School of Public Policy, 1372 Georgia Institute of Technology, Atlanta, Georgia 30332, United States; 4 Institute for Data Engineering & Science, 1372 Georgia Institute of Technology, Atlanta, Georgia 30332, United States

**Keywords:** plastic waste, chemical recycling, pyrolysis, life cycle assessment, circularity, stakeholder engagement, public support

## Abstract

The rapid expansion of the plastic industry has led to significant environmental challenges, prompting the exploration of alternative recycling methods. While mechanical recycling has limitations, chemical recycling, particularly pyrolysis, presents a promising solution. However, it faces contention regarding its environmental impacts and economic feasibility. In this perspective, we analyze both supporting and opposing viewpoints of plastic pyrolysis, highlighting the need for transparent, comprehensive, and measurement-informed life cycle assessments (LCAs) of pyrolysis systems to inform decisions. We also present a case study of literature-reported greenhouse gas (GHG) emissions from pyrolysis-derived ultralow sulfur diesel (ULSD) in the United States, showing that depending on plant capacity and co-product allocation methods, emissions can range from 28% lower to 30% higher than fossil-derived ULSD. Similarly, when viewed as a waste management strategy, net GHG emissions from plastic pyrolysis can range from 220% lower to 60% higher than those from current U.S. plastic waste management practices, depending on system conditions. These findings underscore the variability of results and the need for currently missing, robust, and contextualized LCAs. Finally, we discuss regulatory and social challenges and opportunities for the wider adoption of chemical recycling, emphasizing the critical role of public support in realizing the potential of pyrolysis for a circular economy.

## Graphical abstract

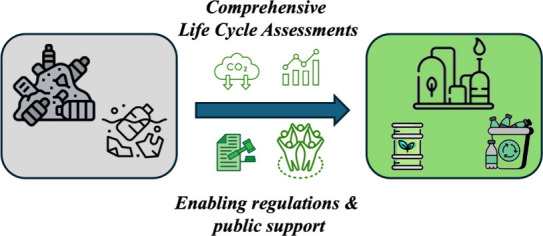

## Introduction

Since the beginning of large-scale industrial plastic production in the 1950s, over 8 billion tons of plastic have been produced,^1^ with production capacities increasing at an annual rate of 4%.^2,3^ The salient attributes of plastics, such as durability, tunability, versatility and low density, have also become the bane of their sustainable use. Globally, an estimated 79% of all plastic produced ends up in landfills, dumps or the natural environment. This includes both managed landfills, typically found in high- and upper- middle-income countries, and unmanaged dumps, which are more prevalent in low- and lower-middle-income countries. Throughout the rest of this text, the term “landfill” refers specifically to managed landfills. The remaining plastic waste is either incinerated (12%) or recycled (9%)^2^ (Figure 1). In addition to the limited availability of land, plastic waste gradually disintegrates to form microplastics, while toxic chemicals can be released.^2^ Consequently, the efficient and sustainable recycling of plastics toward a circular plastic economy has garnered great attention.^2,3^

**Figure 1:**
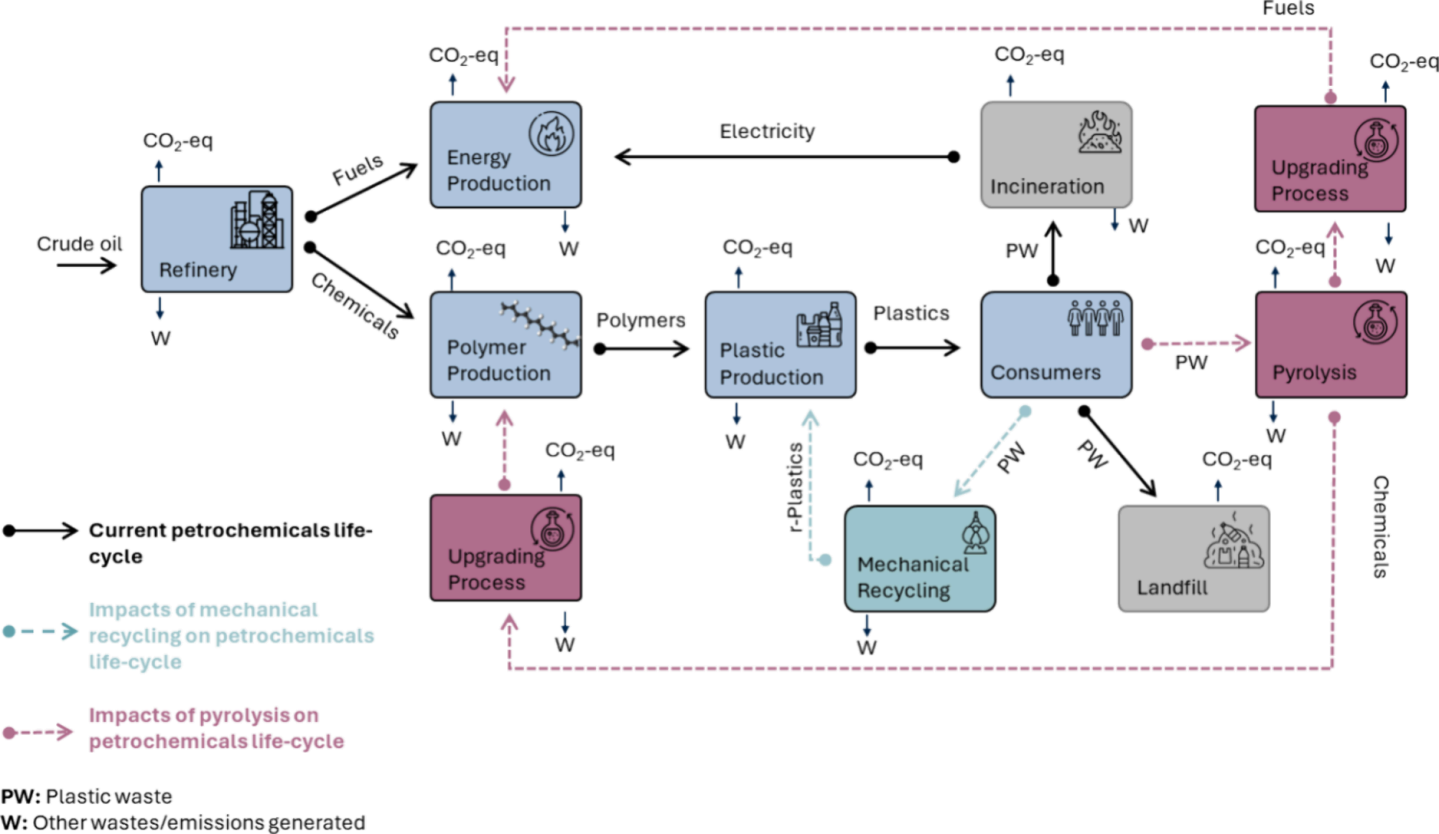
Petrochemical life cycle with and without pyrolysis. The petrochemical life cycle begins with crude oil exploration and extraction, followed by processing at oil refineries to produce fuels and chemicals. Some of these chemicals are used to manufacture polymers, which are then converted into plastic products. Today, the most common plastic waste management practices are landfilling and incineration for electricity production (gray blocks). A small fraction of plastic waste is mechanically recycled (green block and arrows) substituting some of the virgin plastics produced with recycled ones (r-Plastics). Chemical recycling via pyrolysis can provide a more flexible and versatile alternative to mechanical recycling (pink block and arrows). The main products of plastic pyrolysis are diesel oil and naphtha, while fuel gas (noncondensable gases), paraffin wax, and char (solid residue) are common pyrolysis co-products. These products and co-products can be processed further to potentially displace manufacturing of equivalent virgin energy (fuels) or polymer products (chemicals) from fossil resources or can be treated as wastes (W).

Although plastic recycling rates have increased since the 1980s, the lack of significant progress since then is largely attributed to limitations of the most dominant form of recycling, i.e., mechanical recycling, which converts waste plastics into new shapes through mechanical force and heat, typically involving washing, shredding, melting and remolding.^2^ Mechanical recycling leads to degraded material properties, and it requires well-sorted, uncontaminated plastic waste feedstocks, which are frequently not available.^2,4^ Conversely, chemical recycling offers a more flexible approach, able to handle a greater variety of mixed and contaminated plastics as well as multilayer materials. This is achieved by decomposing polymers into monomers or other chemical feedstocks, which can then be repolymerized into materials of superior quality.^2^ Many types of chemical recycling require significant cleaning and sorting to provide feedstocks with well-defined compositions.^4^ If a recycle stream with a defined chemical composition can be obtained, there can be opportunities to maximize value by controlled depolymerization, such as hydrolysis, alcoholysis, or glycolysis of poly­(ethylene terephthalate) (PET).^5,6^ For less homogeneous recycling streams, pyrolysis, the thermally induced breakdown of large polymers into oils and waxes with lower molecular weights in the absence of an oxidizing agent, is a viable chemical recycling alternative. Gasification is another thermochemical process used to convert plastic waste into syngas, which has been compared to pyrolysis in^7,8^ and even combined with pyrolysis^9^ for mixed plastic waste streams. In this perspective, we focus on pyrolysis due to its growing public visibility, promising fuel-like liquid products, and emerging role in circular economy strategies, which have recently attracted significant scientific and societal interest.

Pyrolysis, often mistakenly confused with incineration—which completely combusts the feedstock—enables the conversion of many types of plastics. In particular, pyrolysis reactions of polyolefins are dominated by the cleavage of strong carbon–carbon (C–C) bonds to produce hydrocarbons similar to process streams in oil refineries.^10–12^ However, the formation of chlorinated hydrocarbons and corrosive hydrochloric acid from polyvinyl chloride (PVC), and clogging of process equipment by aromatic acids formed during pyrolysis of PET are problems that need to be addressed by sorting of feedstocks or advanced pyrolysis reactor engineering.^13,14^ Polyolefin pyrolysis is particularly convenient because its products can be almost directly integrated into existing industrial infrastructure, unlike other recycling methods that necessitate significant capital investment for specialized facilities. However, chemical recycling through pyrolysis is still an emerging technology, requiring advances in catalysts, reactor design, and process optimization to become a sustainable and economically feasible solution for processing complex plastic waste.

## Arguments against Plastic Pyrolysis

Several articles in mass media outlets oppose plastic pyrolysis (e.g., refs 15–17 ), often supported by related campaigns from environmental organizations. The most common argument against plastic pyrolysis concerns the energy intensity of the process, mentioning that plastic waste is heated to extremely high temperatures, thereby contributing to greenhouse gas (GHG) emissions, exacerbating climate change.^15,18–24^ It is also noted that pyrolysis facilities emit toxic, cancer-causing pollutants, such as volatile organic compounds (VOCs), particulate matter (PM), and polycyclic aromatic hydrocarbons (PAHs), which can contribute to air pollution and have adverse health effects.^15–22,24–26^ These contaminants can disproportionally affect nearby communities, which mostly comprise marginalized or low-income populations, hence, jeopardizing environmental justice and equity.^16,17^ Furthermore, it is argued that plastic pyrolysis is inefficient and not economically viable, producing end-products that will not be able to compete in price and quality with fossil fuels or virgin plastics.^15,16,18,19,21^ This argument is further reinforced by citing examples of startups and projects that attempted to commercialize chemical recycling in the past decades but failed to achieve industrial-scale production,^18,19,21^ such as the Renewlogy plant in Salt Lake City, Utah, which planned to pyrolyze mixed plastic waste from Boise, Idaho.^16,21^ Moreover, critics contend that pyrolysis’ by-products are contaminated with dioxins and heavy metals, which can potentially pollute soils and water bodies.^15,21,22^ Heavy metals and dioxins are also claimed to be present in the pyrolysis end-products, posing a threat to the environment in the case of combusted fuels,^15,19,22^ or to end-users in the case of recycled plastic materials.^16^ Another commonly cited argument against plastic pyrolysis centers on the fact that it predominantly produces fuels that, when burned, will contribute to GHG emissions, thereby feeding into the cycle of fossil fuel consumption and undermining efforts to reduce carbon footprints and combat climate change, despite being promoted as a recycling solution.^19^ Therefore, some pyrolysis opponents consider it as a competitor to other “reduce-reuse-recycle” efforts, arguing that priority should be given to reducing single-use plastics or promoting mechanical recycling, which could be a more sustainable solution, rather than diverting plastics to pyrolysis.^15,18–20,23^

These concerns are justified given past practices in some industries, and open discussion to address them is valuable. However, it is important to critically evaluate arguments, as many of them are not backed by quantitative analyses. Instead, some rely on arbitrary and anecdotal incidents or the unverified opinions of individuals. Furthermore, assertions often lack transparent evaluations, clear delineations of the processes and steps involved in the analyzed systems and the relevant inputs and outputs considered (defined as “system boundaries” in life cycle assessment (LCA)), as well as fair comparisons with emissions generated from fossil fuel and petrochemical production, or current plastic waste management practices.

Holistic, measurement-informed LCAs, that employ clear and consistent system boundaries, robust and transparent accounting, and appropriate co-product allocation methods are needed. In addition, LCAs should consider a broader range of impact categories beyond climate change and GHG emissions, such as PM formation and ecotoxicity potentials, to provide a more comprehensive view of environmental impacts and to identify potential burden shifting. Such rigorous evaluation, which is currently lacking in the literature, is necessary to provide an accurate assessment of the technology’s potential benefits and drawbacks in comparison to existing practices, while enhancing education and understanding of potential impacts by local communities.

## Systems Analysis for Fuels from Pyrolysis Oils

To assess the environmental impact of chemical recycling of plastics, it is important to place the process and its alternatives in a holistic context. At present, most plastic waste ends up in landfills or is incinerated for energy production.^14^ At the same time, oil reserves are exploited, and crude oil is refined mostly to fuels that are then burned, releasing GHGs. The conversion of waste plastics to fuels would reduce oil exploitation and landfilling of plastics as well as the associated environmental impact of these operations, while meeting needs for fuels that will continue to exist, at least in the immediate future (Figure 1). Of course, a rigorous LCA must compare the energy efficiency and the environmental impacts of conventional fuel production in a refinery with plastics pyrolysis and the required upgrading processes of the pyrolysis oil. Moreover, it has been shown that plastic waste incineration can lead to higher PM emissions than pyrolysis routes for certain waste mixes,^27–29^ while holistic LCA studies of existing waste-management facilities show that even modern incineration facilities coupled with advanced pollution control systems can have significantly improved environmental performance.^30,31^ It will thus be possible to utilize existing technology for sophisticated emissions and solid waste management (e.g., cyclones, wet/dry-scrubbers, filters, activated carbon beds, selective noncatalytic reduction, and advanced process control) in pyrolysis facilities to further reduce environmental impacts well-within regulatory limits.^32^

LCA has been widely utilized to systematically assess the environmental impacts of processes and products across various fields. Through LCA, the environmental aspects and potential impacts across the entire life cycle of a process or product are examined, from the acquisition of raw materials to production, use, end-of-life treatment, recycling, and final disposal.^33^ Over the past decade, numerous LCA studies have been conducted to evaluate the environmental impacts of different plastic waste management schemes and recycling technologies, including plastic pyrolysis for fuel production.^34–48^ These studies vary significantly in scope and assumptions, assessing a range of feedstocks; from single plastics (e.g., polyethylene,^35^ polypropylene,^43,44^ and high-density polyethylene^42,44,48^ ) to mixed plastic^40,41,45–47^ and municipal solid waste^34,36–38^ streams, and evaluating different process configurations that yield different outputs (e.g., fuels, monomers or other chemicals) across various regions (e.g., Asia,^41^ Europe,^36,46^ Oceania,^40^ and United States^34,37,43,47^ ). These differences make comparison among plastic-to-fuel LCA studies and the interpretation of their outcomes extremely challenging. Results are highly sensitive to the analyzed systems (e.g., process sequences and operating conditions, pyrolysis plant sizes, energy mix of particular regions, varying quality and type of used feedstocks and generated products, onsite upgrading capacity and pyrolysis oil upgrading pathways) and the considered methodological factors, such as system boundaries, and co-product handling approaches (e.g., displacement or energy and market allocation).

To facilitate an informed comparison of the environmental performance of pyrolysis of plastic waste for fuel production, we used data from pyrolysis plants operating in the United States, under similar conditions, as reported in two recent studies by the Argonne National Laboratory.^34,38^ Data were categorized based on processing capacity, and co-product handling method.

Three co-product handling methodologies were examined as in refs 34 and 38 for the co-products generated during the production of pyrolysis-derived ultralow sulfur diesel (ULSD), i.e., fuel gas, naphtha and char: energy allocation, market allocation and displacement. Energy allocation refers to distributing environmental impacts among co-products based on their energy content, market allocation assigns impacts based on the relative market value of co-products, and displacement (also referred to as the avoided burden approach) assumes that a co-product displaces an equivalent conventional product, and thus credits the system for the avoided impacts.^33,49^ This modeling choice, can significantly impact the reported emissions.

Furthermore, the system boundaries were adequately adjusted to ensure consistency in the considered steps and the data used from these two studies. Although the use of solvents (e.g., methyl ethyl ketone and toluene) and hydrogen in the separation/upgrading process of pyrolysis oil is reported in the studies, the specific upgrading pathways of pyrolysis oil in each case have not been detailed. Since upgrading pathways of pyrolysis oil is a key factor influencing the environmental outcomes, future assessments would benefit from greater transparency and data availability on this front.

Two systems were considered with different boundaries and functional units, following the rationale of Benavides *et al*.^38^ The first system concerns the “waste plastic to fuel” pathway. The considered system boundary begins with plastic waste collection from municipal solid waste sources and transportation to material recovery facilities (MRFs) for sorting. Sorted plastic waste is then shipped to pyrolysis facilities. Post-industrial waste sent directly to pyrolysis facilities was also considered. All the relevant processing steps prior to plastic waste conversion to pyrolysis oil, including pre-treatment, moisture-content reduction or further sorting, were accounted for. The generated pyrolysis oil undergoes upgrading and/or separation for the production of ULSD. The system boundary ends with the combustion of ULSD during vehicle operation. Impacts from the transportation of collected waste to MRFs and to pyrolysis plants were also considered, where previously unaccounted for. To calculate the transportation burdens, we used ecoinvent v. 3.11 data,^50^ applying the cut-off system model, and considering market activities for freight transport (lorry 7.5–16 metric ton, EURO6 for unsorted plastic waste collection, and lorry >32 metric ton, EURO6 for the transport of sorted plastic waste and post-industrial waste to pyrolysis facilities). We assumed a 10% material loss at the MRF, transportation of unsorted waste to the MRF by refuse trucks (payload of 12.4 tons) over a distance of 80.5 km, and transportation of sorted bales to pyrolysis facilities with diesel trucks, averaging 333 km for medium-scale operations.^34^ The functional unit of this system is 1 MJ of fuel energy, taking into account the energy content of the produced ULSD, i.e., 43.1 MJ/kg. The GHG emissions reported in the two studies were calculated following the IPCC 2014 ^51^ impact assessment methodology. The life-cycle GHG emission burdens reported in Benavides *et al*.,^38^ corresponding to the scenario where the generated fuel gas is combusted to meet only the process heat demand, while any excess fuel gas is sold as an energy product were considered for energy and market allocation and displacement co-product handling methods. A transportation burden of 1.82 g CO_2_-eq/MJ was added to these values taking into account the amounts of feedstock coming from MRFs and the post-industrial waste, as reported in the study and following the IPCC 2014 ^51^ impact assessment method for consistency. The GHG emissions under energy allocation, market allocation and displacement co-product handling methods, as reported by Benavides *et al*.^34^ for different plant capacities were used. The GHG values per MJ of pyrolysis-derived ULSD are presented in the *x* axis of Figure 2.

**Figure 2:**
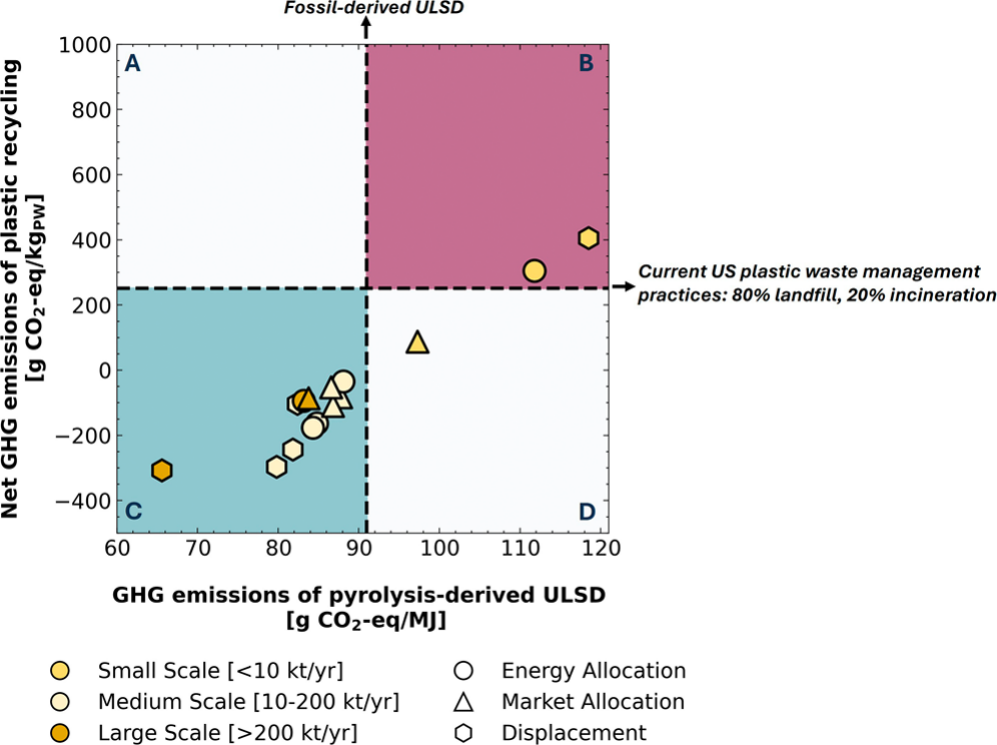
Comparative analysis of GHG emissions associated with pyrolysis of plastic waste. Reported GHG emissions^34,38^ of plastic pyrolysis-derived ULSD (*x* axis) compared to those of fossil-derived ULSD (vertical dashed line); net GHG emissions of recycling plastic waste through pyrolysis to produce ULSD (*y* axis) compared to the net GHG emissions of current plastic waste management practices in the United States (U.S.) (horizontal dashed line). Green plot area (C): Emissions lower than fossil-derived ULSD and current U.S. waste management practices; red plot area (B): Emissions higher than fossil-derived ULSD and current U.S. waste management practices; gray plot areas (A, D): Emissions higher than fossil-derived ULSD or current U.S. waste management practices. Different scales of plastic pyrolysis plants concern different plastic waste processing capacities in kilotonnes/year (kt/yr), with each scale represented by a distinct color. Co-product handling methods are depicted with different shapes, i.e., circles: energy allocation; triangles: market allocation; and hexagons: displacement method.

The second system concerns the “plastic waste management” pathway and assesses the impacts of different plastic waste management approaches. The functional unit of this system is 1 kg of plastic waste (kg_pw_), and the system boundary involves plastic waste undergoing the current waste management practices in the United States (i.e., 80% landfilling and 20% incineration with energy recovery) or being collected, sorted and sent to pyrolysis facilities to produce ULSD (same boundaries as in “waste plastic to fuel” pathway). Relevant transportation steps in both scenarios are included. Assuming that the produced amount of pyrolysis-derived ULSD would displace the same amount of fossil-derived ULSD, the corresponding emissions associated with the production of fossil-derived ULSD were subtracted from those of pyrolysis derived ULSD to calculate the net GHG emissions. A similar rationale has been followed for calculating the net GHG emissions of plastic waste management practices, subtracting the credits of the generated electricity via incineration.^38^ For estimating the net GHG emissions, the energy content and the yield of ULSD as reported in the two studies^34,38^ were considered. The net GHG emissions associated with the current plastic waste management practices in the United States were considered as reported in Benavides *et al*.^38^ The net GHG values per kg of plastic waste are presented in the *y* axis of Figure 2. GHG emissions of production and use of plastic pyrolysis-derived ULSD (“waste plastic to fuel” pathway) were compared to those of fossil-derived ULSD, and net GHG emissions of recycling plastic waste through pyrolysis to produce ULSD (“plastic waste management” pathway) were compared to the net GHG emissions associated with current plastic waste management practices in the United States (Figure 2). Fossil-derived ULSD emits 91 g CO_2_-eq per MJ of generated energy (vertical dotted line), while the current plastic waste management practices in the United States generate 250 g CO_2_/kg_PW_ (horizontal dotted line).^34,38^

Given the different plant processing capacities and the selected co-product handling methods, the GHG emissions of pyrolysis-derived ULSD can range from 28% lower (∼66 g CO_2_-eq/MJ) to 30% (∼119 g CO_2_-eq/MJ) higher than those from fossil-derived ULSD. Similarly, recycling plastic waste via pyrolysis can result in net GHG emissions ranging from approximately 220% lower (∼ −308 g CO_2_-eq/kg_pw_) to 60% higher (∼305 g CO_2_-eq/kg_pw_) than those of current landfilling and incineration practices in the United States. This variability in GHG emission values indicates the need for robust, contextualized LCAs to support informed decision-making.

Data points in the green, lower left region (C) of Figure 2 showcase processes that exhibit reductions in GHG emissions compared to both fossil-derived ULSD and conventional waste management practices. In contrast, the red, upper right region (B) represents processes with GHG emissions that exceed those of business-as-usual scenarios. In cases where the GHG emissions from the production and use of pyrolysis-derived ULSD are lower than those of fossil-derived ULSD (Figure 2, region C), the corresponding net GHG emissions associated with plastic waste recycling become negative. This is because the avoided emissions from displacing fossil-derived ULSD exceed the emissions generated by the pyrolysis-derived fuel.

As observed, plants with lower processing capacities generate higher GHG emissions, regardless of the co-product handling method, demonstrating the effect of economies of scale on the environmental impacts of the pyrolysis process. Economies of scale are also anticipated to have a positive effect on the economic viability of recycling processes. According to Benavides *et al*.,^34^ larger-scale pyrolysis plants not only achieve lower GHG emissions but also exhibit significantly reduced fossil energy and water consumption. These improvements in resource efficiency can result in meaningful cost savings, particularly in terms of energy procurement and wastewater treatment, which can be substantial operational expenses. Furthermore, higher-capacity facilities can better absorb fixed capital and maintenance costs, improve equipment utilization, and benefit from more competitive logistics and feedstock procurement strategies.^52,53^ As such, scaling up pyrolysis operations can enhance both environmental performance and economic feasibility, making large-scale plants more attractive from both sustainability and investment standpoints.

The boundaries of the environmentally friendly region defined by the horizontal dotted line, representing the net GHG emissions of the current waste management practices in the United States, i.e., the baseline scenario, are not fixed and may shift over time. This baseline scenario is influenced by the shares of landfilling and incineration, as well as the carbon intensity of the electricity displaced by the incineration of waste plastic. The carbon intensity of displaced electricity depends on the local energy mix (e.g., coal or renewables), which may vary by region and change over time. Landfilling of plastic waste results in negligible GHG emissions. Therefore, the net GHG emissions of the baseline scenario (80% landfilling – 20% incineration) would increase with greater reliance on incineration, which produces direct emissions (∼2.5–3 kg CO_2_-eq per kg_pw_ being incinerated without considering credits of replaced energy^14,38,49^ ), while offering partial offsets through electricity generation. However, the effectiveness of this offset is decreasing as the share of carbon-neutral electricity sources grows. The GHG intensity of electricity generation in the United States has dropped significantly over the past decades, reaching about 0.1 kg CO_2_-eq/MJ in 2023 - a reduction of almost 43% compared to 2005 levels.^54^ This decline is attributable to several factors, such as the transition from coal to gas and the increased power generation from renewables. As the carbon intensity of the grid drops, the GHG offset associated with incineration-generated electricity diminishes. In a future scenario, where landfilling becomes obsolete in the United States, and incineration for energy generation is a more prevalent practice, while the energy mix is greener, the horizontal dotted line in Figure 2 would shift upward, since this would lead to higher emissions from the baseline waste management scenario. This shift would indicate that even pyrolysis plants with lower capacities (<10 kt/yr) could offer improved environmental performance compared to conventional plastic waste management practices. Therefore, the outlook for the United States suggests that the relative advantage of plastic-to-fuel scenarios through pyrolysis over the baseline, is likely to strengthen over time.

## Potential for Products Other than Fuels

While arguments for and against the use of plastics pyrolysis products as transportation fuels (or carbon-based transportation fuels in general) can be made, it is important to consider the potential of fuels from pyrolysis oils as a transitional solution. As electric vehicles and green electricity continue to increase their market shares, there will be a decreasing demand for these fuels. Consequently, it will be critical to develop processes that can channel the products from plastics pyrolysis into chemicals and materials, particularly commodities that are currently co-produced with fuels in refineries.^55^ Otherwise, societies would risk supply shortages when current integrated petrochemical supply chains are starting to decline.

Certain product cuts from pyrolysis oils could be refined to high-value waxes and lubricants. In the presence of a suitable catalyst, waxy products from pyrolysis can be further cracked into oils and other petrochemical intermediates (e.g., propylene) using existing fluid catalytic cracking (FCC) units.^56,57^ Additionally, steam crackers can convert a wide range of hydrocarbon feeds into olefins with sizable markets, if certain heteroatoms are removed to a sufficient extent.^58^ These olefins can be used as feedstock in the petrochemical industry to produce a range of products including plastics and synthetic fibers.

In certain cases, pyrolysis processes can provide high yields of monomers even from poly­(olefin)­s. Poly­(styrene) and poly­(methyl methacrylate) have sufficiently low ceiling temperatures (i.e., 310 and 220 °C, respectively)^59^ to be depolymerized to monomers by pyrolysis at reduced pressures. Flash pyrolysis, a process performed at high temperatures (close to 1,000 °C) and with short vapor residence times (less than 250 ms), can yield up to 50 wt % ethylene monomers from poly­(ethylene) waste.^10^ The production of a wide range of materials from waste plastics has also been reported, including carbon nanotubes, charcoal, and carbon black, which can be used to reinforce rubber and plastics.^56,57^ For example, up to 25 wt % of waste plastic can be converted into carbon nanotubes, which are a higher-value though lower volume product than fuels.^56^

To match the scale at which waste plastics are discarded, it is critical to develop environmentally benign and economically viable processes that can supply the markets for major commodities, such as ethylene, propylene, benzene, toluene, and xylene (BTX). A recent study showed, that BTX can be produced cost-competitively and with reduced supply chain energy requirements from plastic pyrolysis, but several processing steps would need to be improved to avoid increased GHG emissions.^60^ Since a significant portion of waste plastics comprises composites of multiple materials, a feedstock-tolerant technology like pyrolysis could reduce the need for sorting and purification, while maximizing the fraction of the waste stream that can be recycled.

## Challenges and Opportunities on the Wider Adoption of Pyrolysis and Chemical Recycling

To facilitate the broader adoption of chemical recycling, including pyrolysis, enabling regulations and heightened public awareness are needed. Suppliers of pyrolysis-derived products seeking market access need additional regulatory incentives to ensure long-term price stability and credit for enhanced material recovery.^13^ Since 2017, 24 U.S. states have passed laws to promote the chemical recycling of plastics.^61,62^ These state laws offer regulatory stability to companies investing in new chemical recycling plants. They often share key provisions, such as classifying chemical plastic recycling facilities as manufacturing plants, rather than waste-handling facilities. Such classification can carry important regulatory and economic advantages, enabling chemical recycling plants to qualify for government financial incentives that apply for new manufacturing facilities, including state and local tax breaks or access to government bonds to support construction.^63,64^

U.S. state laws vary on whether converting plastics to fuels is considered recycling.^63,64^ Some states (e.g., Iowa^65^ and Ohio^66^) allow chemical recyclers to produce fuels and plastic feedstocks, while others (e.g., Kentucky^67^ and Arkansas^68^) exclude waste-to-energy processes from their recycling definitions to promote plastic-to-plastic recycling. Additionally, some state laws are less specific on this topic indicating that products of chemical recycling should have commercial value either as raw materials or finished goods (e.g., West Virginia^69^ and Mississippi^70^). The spread of state laws on chemical recycling is contributing to a multisector effort seeking regulatory relief from the U.S. Environmental Protection Agency (U.S. EPA).^63,64^ Industry stakeholders are requesting that the U.S. EPA not subject pyrolysis and gasification units to the stringent Clean Air Act regulations that apply to incinerators that combust solid waste. While the U.S. EPA initially proposed to accommodate this request, the proposal remains unfinalized and is currently open for public comment to ensure public health protection and consistency with measures and controls for similar manufacturers and facilities.

Meanwhile, some states are opposing chemical recycling practices.^62,63,71^ For example, Maine introduced a bill in 2023 ^72^ declaring that chemical recycling does not constitute recycling but solid waste processing. A failed bill in Rhode Island^73^ aimed to prohibit the construction of chemical recycling facilities, while Vermont^74^ and Massachusetts^75^ have introduced bills that seek to ban chemical recycling practices entirely. Political pressure is mounting given this controversy which highlights the need for adopting federal regulations.

Despite relevant laws, public support is crucial for the development of new chemical recycling facilities. For instance, the Macon-Bibb County Industrial Authority considered offering $500 million in industrial revenue bonds for a $680 million plant in Georgia, which passed a chemical plastics recycling law in 2018.^64,76^ However, due to public opposition, the project was canceled.^76^ This example highlights the significant impact of community support on the adoption of chemical recycling. Therefore, it is highly important to co-design such facilities together with the local stakeholders, so as to ensure reduced environmental impacts compared to counterfactual scenarios and prioritize environmental justice and community well-being. To foster well-informed and constructive discussions and to dispel unfounded fears, it is imperative to rely on well-justified, transparent scientific evidence and a complete cradle-to-grave/cradle analysis of the recycling process and any processes that it intends to replace, as opposed to the analysis of arbitrarily selected subsystems.

To broaden the appeal of pyrolysis and other chemical recycling technologies, it is essential to raise public awareness of the benefits of converting post-consumer plastics into new plastics or fuels. Public engagement in discussions about encouraging and funding pyrolysis hinges on perceptions of its societal benefits. Embracing pyrolysis offers an opportunity to divert significant amounts of contaminated plastic waste from landfills, but it also requires a shift in public behavior toward recycling plastics after use. Behavioral science-based information campaigns have shown promise in increasing awareness of Extended Producer Responsibility (EPR) initiatives, which mandate companies to account for the end-of-life environmental costs of their products to reduce packaging waste and increase recycling volumes. These campaigns can include non-monetary interventions to enhance recycling efforts,^77^ improve plastic waste sorting,^78^ and promote resource conservation and plastic waste management through modern approaches including use of artificial intelligence for discovery of consumer preferences.^79–81^ Additional interventions focused on fostering social acceptance of chemical recycling technologies, like those employed for renewable energy technologies, and incentives designed under EPR laws are crucial. Furthermore, providing direct evidence on potential trade-offs between increased material recovery and generated emissions with public health implications is needed. Finally, we argue that support for chemical recycling should not be a distraction from reduce-reuse-recycle principles. Instead, chemical recycling technologies could complement existing efforts by addressing hard-to-recycle materials, while the core hierarchy of waste reduction must always remain our priority and be considered in future life cycle and market analyses.

## Concluding Remarks

Addressing climate change and plastic waste issues requires a multifaceted approach. While reducing the use of virgin plastics and promoting reuse should be the primary priority, plastic pyrolysis emerges as a promising transitional solution which invariably involves public choice. To enable sustainable and circular transitions, it is crucial to incorporate diverse perspectives, including those critical of chemical recycling, into a well-informed discourse. As scientists, it is our responsibility to listen to the needs and concerns of all stakeholders and communities affected by such interventions. We must strive to design environmentally friendly processes and provide integrated LCA studies addressing concerns and measuring impacts accurately to support informed decisions. Importantly, transparent, measurement-informed LCAs tailored to specific geographies, technologies, and policy contexts are needed. LCA needs to be performed from cradle-to-grave/cradle perspectives to avoid misguided conclusions due to arbitrarily selected system boundaries. By adopting a holistic approach incorporating social, environmental, and economic aspects, and by harnessing the potential of technologies like pyrolysis, we can pave the way for a more sustainable future, tackling plastic waste while advancing environmental stewardship.
